# Dog ownership, dog behaviour and transmission of *Echinococcus* spp. in the Alay Valley, southern Kyrgyzstan

**DOI:** 10.1017/S0031182013001182

**Published:** 2013-08-28

**Authors:** FREYA VAN KESTEREN, ALEXANDER MASTIN, BERMET MYTYNOVA, ISKENDER ZIADINOV, BELGEES BOUFANA, PAUL R. TORGERSON, MICHAEL T. ROGAN, PHILIP S. CRAIG

**Affiliations:** 1 Cestode Zoonoses Research Group, School of Environment and Life Sciences, University of Salford, M5 4WT Salford, UK; 2 Kyrgyz Veterinary Research Institute, Togolok Moldo 60, Bishkek, Kyrgyzstan; 3 Section of Veterinary Epidemiology, University of Zürich, Winterthurerstrasse 266a, CH-8057 Zürich, Switzerland

**Keywords:** *Echinococcus granulosus*, *Echinococcus canadensis*, *Echinococcus multilocularis*, Kyrgyzstan, domestic dogs, demography, behaviour

## Abstract

Echinococcosis is a re-emerging zoonotic disease in Kyrgyzstan, and the incidence of human infection has increased substantially since the collapse of the Soviet Union in 1991. Domestic dogs are hosts of *Echinococcus* spp. and play an important role in the transmission of these parasites. The demography, ecology and behaviour of dogs are therefore relevant in studying *Echinococcus* spp. transmission. Dog demographics, roles of dogs, dog movements and faecal environmental contamination were assessed in four rural communities in the Alay Valley, southern Kyrgyzstan. Arecoline purge data revealed for the first time that *E. granulosus, E. canadensis* and *E. multilocularis* were present in domestic dogs in the Alay Valley. Surveys revealed that many households had dogs and that dogs played various roles in the communities, as pets, guard dogs or sheep dogs. Almost all dogs were free to roam, and GPS data revealed that many moved outside their communities, thus being able to scavenge offal and consume rodents. Faecal environmental contamination was high, presenting a significant infection risk to the local communities.

## INTRODUCTION

Echinococcosis is a neglected zoonotic disease (World Health Organization, 2010) caused by infection with the larval stage (metacestode) of tapeworms within the genus *Echinococcus* (Eckert and Deplazes, 2004). The most common types of echinococcosis are cystic and alveolar which are caused by *E. granulosus* and *E. multilocularis,* respectively (World Health Organization/World Organisation for Animal Health, 2001). The life-cycles of *E. granulosus* and *E. multilocularis* involve two mammalian hosts. The adult cestode inhabits the small intestine of a definitive host (usually a canid) and produces eggs which are released into the environment (Eckert and Deplazes, 2004) and may then be ingested by an intermediate host. In the case of *E. granulosus* the intermediate host is usually a sheep, but may include other herbivore species (Sweatman and Williams, 1963; World Health Organization/World Organisation for Animal Health, 2001). In the case of *E. multilocularis*, small mammals, including voles (e.g. *Arvicola* spp., Hofer *et al.* 2000), pika (*Ochotona* spp., Schantz *et al.* 2003), and Tibetan hare (*Lepus oiostolus*, Xiao *et al.* 2004) may act as intermediate hosts. If the intermediate host is consumed by a definitive host, the cycle is complete. Humans may also inadvertently ingest eggs expelled by the definitive host and develop cystic or alveolar echinococcosis (Deplazes and Eckert, 2001).

In Asia, echinococcosis is a serious public health concern in several areas including the Tibetan Plateau (Budke *et al.* 2005), central China (Craig *et al*. 1992), and Mongolia (Ebright *et al.* 2003; Ito *et al.* 2010). There is concern that echinococcosis is re-emerging in Central Asia following the collapse of the Soviet Union (Torgerson *et al.* 2006). In Kyrgyzstan, the annual human surgical incidence of cystic echinococcosis has increased dramatically, rising from about 5 cases per 100 000 to nearly 20 cases per 100 000 between 1991 and 2002 (Torgerson *et al.* 2006). Alveolar echinococcosis is also thought to be increasing in Kyrgyzstan (Torgerson *et al.* 2010; Usubalieva *et al.* 2013).

Although several species of wild canids may be hosts for *E. granulosus*, e.g. grey wolves, *Canis lupus* (Abdybekova and Torgerson, 2012) and *E. multilocularis*, e.g. red fox, *Vulpes vulpes* (Ziadinov *et al.* 2010), infected domestic dogs, *Canis familiaris*, are considered to pose the greatest risk of human infection (Budke *et al.* 2005). Domestic dogs are hosts for a number of zoonotic pathogens, and due to their close association with people they may be sources of human infections (Macpherson, 2005). The demography, ecology and behaviour of dogs are therefore relevant in studying diseases that may be spread by them, and describing the dog population in a community may help to assess transmission potential, zoonotic risks and optimization of intervention programmes (Butler and Bingham, 2000). This concept has been recognized in studies relating to rabies (Perry, 1993; Butler and Bingham, 2000; Kitala *et al*. 2001; Macpherson, 2005), but has to date rarely been applied to studies on echinococcosis (but see Vaniscotte *et al.* 2011). Here we aim to determine the presence of *Echinococcus* spp. in domestic dogs in four communities in southern Kyrgyzstan. We further aim to characterize the domestic dog population in these communities by describing their demographics, roles, husbandry and roaming behaviour, as well as the levels of environmental contamination with dog faeces. In doing so, we aim to understand better the role of dogs in *Echinococcus* spp. transmission in these communities.

## MATERIALS AND METHODS

### Study site

The Alay Valley is located in the south of Kyrgyzstan, and covers most of the Osh oblast. It is located at an altitude of approximately 3000 m a.s.l. and is bordered by the Pamir Mountains to the south (on the border with Tajikistan), and the Alay Mountains to the north (Wikipedia, 2013). Based on a cluster of human AE cases derived from hospital records reported by Usubalieva *et al.* (2013), we selected four rural communities in the Alay Valley for a study of canine echinococcosis, namely Taldy Suu (39·70°, 72·98°) Sary Mogol (39·68°, 72·89°), Kara Kavak (39·66°, 72·72°) and Kashka Suu (39·64°, 72·67°).

### Household questionnaires

Detailed questionnaire-based surveys were carried out in May 2012. Questionnaires were designed using WHO guidelines (World Health Organization and World Society for the Protection of Animals, 1990). Questions were asked about the age, sex and source of dogs, as well as their reproductive status (if female). Questions were also asked about the role of the dog, i.e. pet, guard, sheep dog or other. Further questions were included about the diet of the dog, and whether it was ever tied up. On a subsequent visit in October 2012, shorter questionnaires were employed to ask households if they still owned the previously registered dogs and if any new dogs had been acquired. If the previously registered dogs were no longer present, the reason for this was asked. Not all questions were answered by all respondents, so that numbers reported are at times less than the total number of dogs registered. All questionnaires were administered in Kyrgyz by native speakers (BM and IZ).

### Faecal quadrats to assess environmental contamination

ArcGIS was used to create shapefiles of the approximate boundaries of the four villages (based on imagery from the SPOT5 satellite, Google Maps, 2012). Within each of these four areas, 17 random points were generated which were used to define one corner of each quadrat. If the point fell in an inaccessible location (e.g. a house) the nearest possible point was taken. The direction of the quadrat was usually determined by the surrounding buildings, fences, etc. Where there was enough space for the 50×50 m quadrat to be done facing several directions, the second hand on a watch was used to determine the direction. Where it was not possible to measure out 50×50 m due to the presence of buildings, smaller areas were measured and the size was recorded. Quadrats were searched for faeces by slowly pacing up and down whilst looking at the ground. Canid faeces were identified by their size and shape by a researcher experienced in the identification of canid faeces (FvK). In all likelihood, faeces found in villages were from domestic dogs although some may have been from foxes and possibly wolves. As dogs, foxes and wolves are all hosts for *Echinococcus* spp. and therefore all pose an infection risk to humans, no effort was made to distinguish between these using host PCR. The faecal density was calculated as the number of faeces/100 m^2^. All quadrats were performed by the same researcher, and the same 68 quadrats were searched for faeces in May and in October 2012.

During each visit, four quadrats were selected in each village using a random number generator in Microsoft Excel^®^ (Microsoft, Redmond, WA). In all four quadrats, one third of faecal samples (or at least six, if the total number of faeces was less than 18) were selected by sequentially ordering the samples prior to using an Excel^®^ random number generator. Since these samples were collected from the ground we cannot be sure that samples collected were from different individual dogs. Sub-samples of each of these were stored in 0·3% PBS Tween buffer with 10% formalin and 95% ethanol for coproantigen ELISA and coproPCR analysis, respectively and were shipped to the University of Salford at ambient temperature.

### Coproantigen ELISA

All faecal samples collected were analysed using a genus-specific coproantigen ELISA. This allowed us to detect those samples that contained *Echinococcus* spp. antigens before using coproPCR (see below) to identify whether the samples were positive for *E. multilocularis* or *E. granulosus* sensu lato. Coproantigen ELISA allows for rapid analysis of large numbers samples and can detect pre-patent infections (Fraser and Craig, 1997), and can be recommended as a primary diagnostic tool followed by PCR (e.g. Eckert and Deplazes, 2004). Samples were first stored at −80 °C for a minimum of 5 days in order to kill any infective eggs of *Echinococcus* spp. (World Health Organization/World Organisation for Animal Health, 2001). Samples were then defrosted and homogenized with wooden spatulas, shaken and centrifuged at 2500 rpm for 5 min using an Eppendorf centrifuge 5804. Supernatants were decanted into Bijoux tubes and stored at −20 °C until used for analysis. A genus-specific sandwich ELISA, using the protocol described by Allan *et al*. (1992) and Craig *et al*. (1995), was used to test for *Echinococcus* spp. coproantigen. Supernatants of two known positives (an arecoline purge-positive sample from Kara Kavak and an antigen-spiked sample, spiked 1:100 with *E. granulosus* whole worm extract) were used as positive controls throughout. Two known negatives from non-endemic and endemic areas (Manchester, UK and the Falkland Islands) were also included as negative controls.

### CoproPCR

CoproDNA was extracted from 1 g of dog faeces (weighed using a balance accurate to 0·01 g) using QIAamp^®^ DNA Stool Mini Kit following the manufacturer's instructions. The DNA retrieved was used to test for *E. granulosus* using two sets of primers. Initially, samples were tested for *E. granulosus* G1 (common sheep strain) using highly specific ND1 primers to amplify a species-specific 226 bp fragment (Boufana *et al.* 2013). However, because the strains of *E. granulosus* present in this area are unknown, another protocol was used to detect *E. granulosus* (sensu lato) by amplifying a 269 bp tandem repeat region (Abbasi *et al.* 2003) using modifications described by Boufana *et al.* (2008). DNA samples were also tested for *E. multilocularis* using PCR-specific primers (Boufana *et al.* 2013) to amplify a 207 bp fragment within the ND1 mitochondrial gene. Negative controls (PCR grade water) were included throughout. A Stratagene Robocycler (La Jolla, CA) was used for all cycling profiles and the PCR products were separated by electrophoresis using a 1·5% (w/v) agarose gel (Bioline) in 1× Tris-Borate-EDTA buffer (Severn Biotech, Kidderminster, UK) at 110 V, stained with gel red DNA dye (Cambridge Biosciences, Cambridge, UK), and visualized using Syngene G:Box gel documentation system (Cambridge Biosciences).

### Arecoline purges

Twenty dogs (16 from Kara Kavak and 4 fromTaldy Suu) were voluntarily brought in by their owners and dosed with a 0·4% solution of arecoline hydrobromide in water (7 mg arecoline/kg body weight) and were restrained safely by their owners until they purged. The purges were examined in the field using a handheld magnifying glass and scored for presence/absence of *Echinococcus* spp. and *Taenia* spp. based on gross morphology by an experienced fieldworker (PSC). Subsamples of these purges were stored in 0·3% PBS Tween buffer with 10% formalin and 95% ethanol for coproantigen and coproPCR analysis, respectively and shipped to the University of Salford at ambient temperature.

### Dog tracking

A total of 40 dogs (11 from Sary Mogol, 14 from Taldy Suu, 12 from Kashka Suu and three from Kara Kavak) were fitted with iGotU^®^ GPS trackers. The iGotU^®^ unit is a GPS tracker that can record GPS positions at programmed intervals (www.i-gotu.com). These units were attached to regular dog collars using ziplock bags and adhesive tape. The accuracy of the GPS units was validated both by leaving units in set locations (stationary recording) and by moving units along a path (dynamic recording). These activities were undertaken in both the Alay Valley and in relatively sparsely built-up areas in the United Kingdom (adjacent to South Park, Macclesfield for stationary recording and Peel Park, Salford for dynamic recording). In both cases, a Garmin^®^ GPS60 unit was used for comparison.

Dogs were selected for collaring in the field during dog registration. Selection could not be completely random as only those dogs present and tame enough to be handled were selected. Although an effort was made to track each selected dog for 24 h this was not always possible due to field logistics and limitations in battery life. In addition, a number of GPS trackers could not be retrieved. Dogs were tracked for between 1·5 and 47 h (mean = 20 h, s.d. = 9 h), and trackers were set to record GPS positions every 5 min, with between 25 and 380 positions recorded per dog (mean = 156, s.d. = 81). Dogs were recorded for a total of 787 h, with a total of 6256 GPS points recorded. However, dogs with fewer than 50 points recorded (*n* = 3) were removed from further analysis, leaving a total of 37 dogs. Of these 37, 26 were male (SM = 7, TS = 11, KS = 5, KK = 3) and 7 were female (SM = 4, KS = 3). For four dogs the sex was not recorded.

### Data analysis

Data were analysed and figures were made using R statistical software version 2.15.2 (R Development Core Team, 2012). In order to analyse the quadrat data, the glme function in the lme4 package (Bates *et al*. 2012) was used to create a Poisson-normal generalized linear mixed effects model. This was used to model the number of faeces within each quadrat and investigate the effect of village and date of visit. The log area of the quadrat was included as an offset variable in the model and the quadrat ID was included as a random effect in order to account for overdispersion. Models were created including both village-specific and overall random effects and were compared using a likelihood ratio test. In order to assess for overdispersion in the final model, the ratio of the sum of squared Pearson residuals to the residual degrees of freedom was calculated with a value of greater than one used to suggest overdispersion.

The accuracy of the iGotU units used for monitoring dog movements was tested using both stationary and dynamic recordings. The accuracy of the stationary units was estimated by calculating the distance recorded by the units from the true location (as determined by the Garmin^®^ unit), using the ‘Hub Distance’ tool in the MMQGIS add-on (http://michaelminn.com/linux/mmqgis) for Quantum GIS 1.8.0 (Quantum GIS Development Team, 2012). For the dynamic data, all points were matched to the nearest time point recorded by the Garmin^®^ unit and the distance between these points was estimated using the ‘Hub Lines’ tool in the MMQGIS add-on for Quantum GIS.

Analysis of dog movements was conducted in order to characterize both the size of the ‘home range’ of these animals and the total distances travelled from the household. The R package ‘adehabitatHR’ (Calenge, 2006) was used for the estimation of home range size. For this, the characteristic hull polygon (CHP) method first developed by Downs and Horner (2009) was used due to the recognized limitations of the usual minimum convex polygon (MCP) and kernel density methods which have been used historically. The total areas of these home ranges were estimated using Quantum GIS 1.8.0 and exported to R for further analysis.

The ‘Hub Lines’ tool in the MMQGIS add-in for Quantum GIS was used to estimate the minimum distance between each relocation point and the start point for each animal. Violin plots were created using the ‘vioplot’ package’ (Adler, 2005), and confidence intervals for the village-specific median distance travelled (calculated from the median distances travelled per dog) were bootstrapped from the data using the ‘boot’ package (Canty and Ripley, 2012) with 1000 replications. Researchers conducting household surveys carried Garmin GPS units with tracking mode enabled. These data were analysed using the MCP method in the Geoprocessing Tools of Quantum GIS in order to identify the boundaries of the villages under study. These were then used to estimate the number and proportion of dog relocations which were outside the village boundaries. Differences in home ranges and median distance moved (per dog) between male and female dogs were compared using the Wilcoxon rank sum test.

## RESULTS

### Dog owner questionnaire data

A total of 644 households were registered in the four communities, with a combined population of 3677 people (Table 1). Questionnaire data revealed that between 38·0 and 74·4% of households in the four communities had at least one dog, with a total estimated dog population of 393, or 1 dog for every 9·36 people (although this does not include the total dog population in Kashka Suu, where only a sample of dogs was taken).

**Table 1.** Characteristics of the populations under investigation

	Village			
	Sary Mogol	Taldy Suu	Kashka Suu	Kara Kavak
Number of households registered	368	125	86	65
Total number of people	2173	588	518	398
Total number of dogs reported	178	119	50	46
Percentage of households with at least one dog	38%	74%	51%	52%
Total number of dogs registered	155	115	49	38

**Fig. 1.**
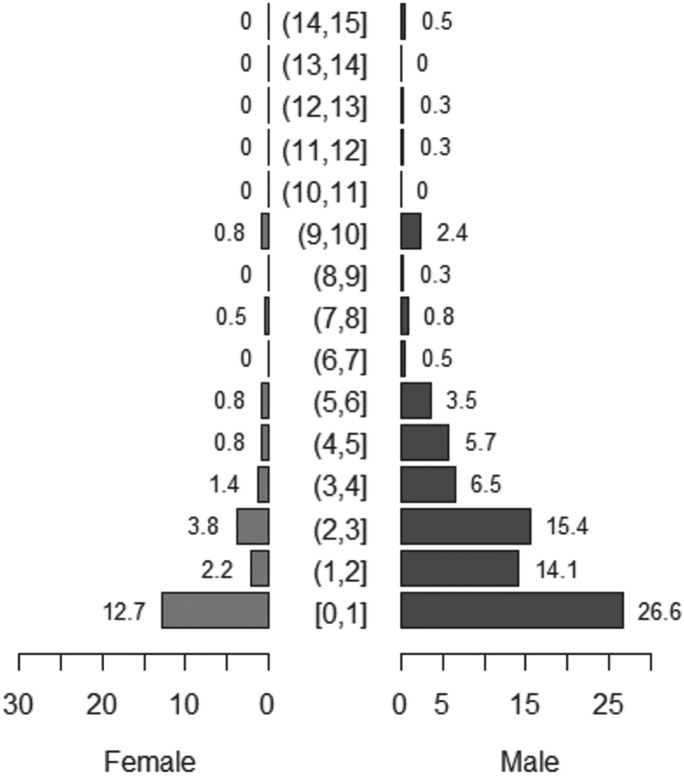
Population pyramid for all dogs sampled in May 2012 (*n* = 383). Numbers represent total proportion of dogs in each age and sex group.

Reported dog ages ranged from 2 weeks to 15 years, with a median age of two years. Males represented around 77% of the total dog population. Fig. 1 shows the population pyramid for all dogs sampled.

**Fig. 2.**
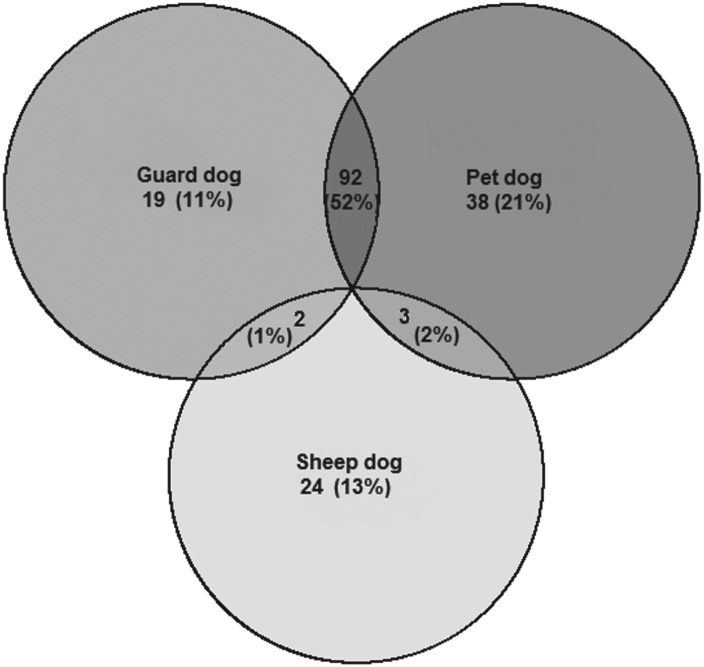
Euler diagram of the reported uses of dogs registered in the study. Numbers represent total number of dogs in each category.

People in all four communities believed there were un-owned ‘stray’ dogs in their village (SM: 45·9%, TS: 8·8%, KK: 4·6%, KS: 14·0%) although few people who reported a stray dog population had any idea of the size of it. We registered 357 dogs and asked questions about their role and management. Around 75% of dogs for which this question was answered were described as pets, although many of these were also described as guard dogs, as shown in Fig. 2. Dogs were never reported as being kept as a source of meat, and consumption of dog meat is not customary in these mostly Muslim communities.

**Fig. 3.**
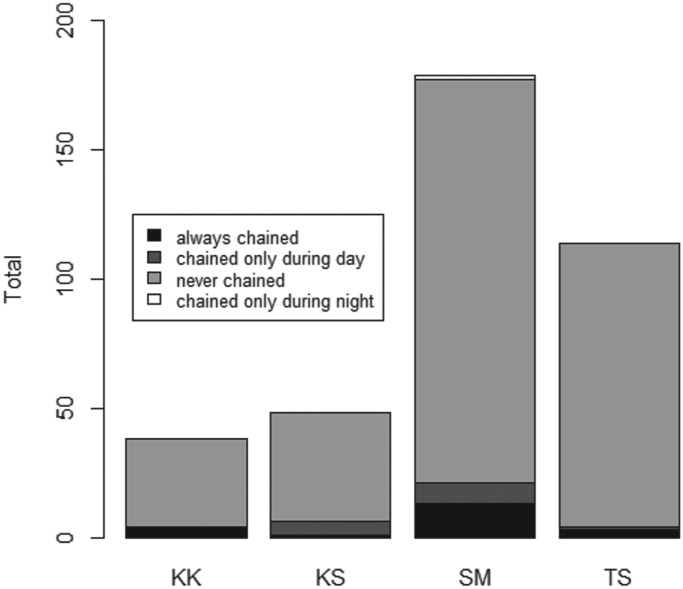
Frequency of dog restraint in the study villages.

The proportion of dogs which were never restrained and free to roam at will was higher in Taldy Suu (110/114 = 96%) than in the other villages (232/265 = 88%, Chi square *P* = 0·01). Of the remaining dogs, most were always chained (see Fig. 3).

**Fig. 4.**
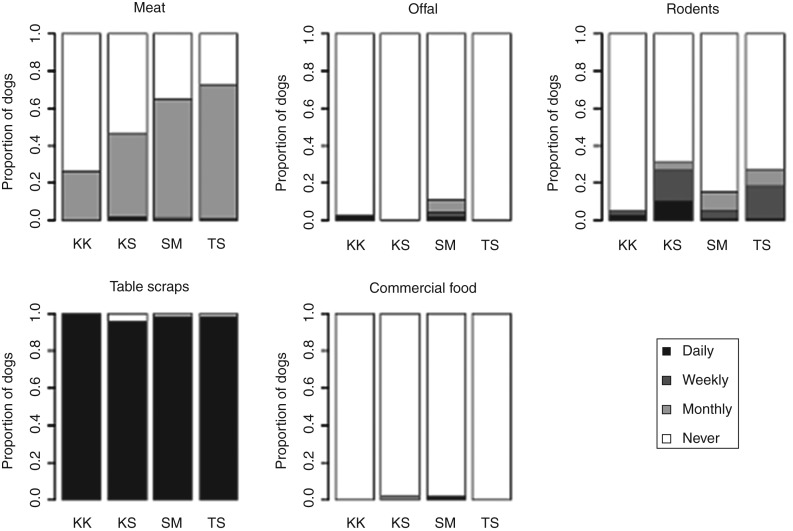
Proportion of dogs fed different food types and reported frequencies of feeding.

Dogs were all fed by household members and were most often fed table scraps, although offal was also reported to be commonly fed (Fig. 4). Dogs were rarely observed eating rodents, although this was occasionally reported – especially in Kashka Suu and Taldy Suu (Fig. 4).

In May 2012, a total of 222 owned dogs were thought to be present in Sary Mogol; 141 in Taldy Suu; and 41 in Kara Kavak. Based on crude estimates of numbers of households from remote sensing data, it is assumed that around 25% of households in Kashka Suu were visited – suggesting a total owned dog population of around 200 in this village. In October, the owned dog population was reduced in all three of these villages – to 121 dogs in Sary Mogol; 126 in Taldy Suu; and 36 in Kara Kavak. The majority of this difference resulted from the loss of dogs (usually either as missing dogs, through accidental death or by culling), although some dogs had moved to mountain pastures. Between the two visits, a total of 52 new dogs (usually puppies) were obtained in Sary Mogol, 36 in Taldy Suu and four in Kara Kavak (although it should be noted that in Kara Kavak, only those households which previously had dogs were revisited in October 2012). Using the data from the census villages of Sary Mogol and Taldy Suu, this suggests that per owned dog present in May, the rate of removal over the 5 months between visits was around 0·7 in Sary Mogol and 0·4 in Taldy Suu, whereas the replacement rate was around 0·2 in Sary Mogol and around 0·3 in Taldy Suu. It is also important to note that the estimates for Taldy Suu were made prior to the second visit of the person responsible for dog culling, whereas those for Sary Mogol were made after this visit.

### Quadrats for assessing faecal environmental contamination

**Fig. 5.**
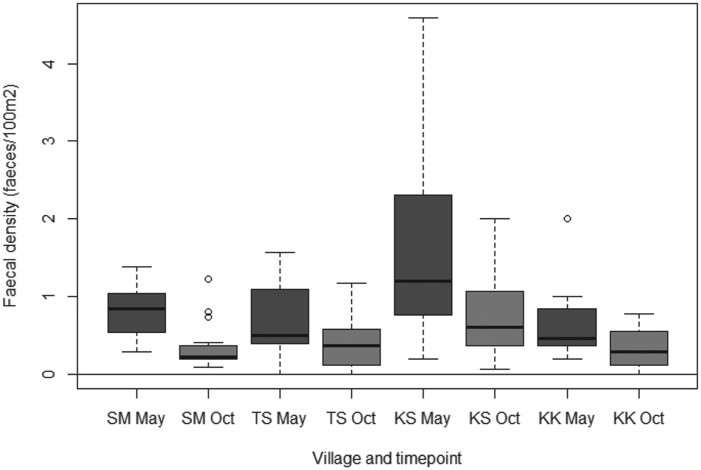
Canid faecal densities amongst the different villages visited in May and October 2012 (KK = Kara Kavak, KS = Kashka Suu, SM = Sary Mogol, TS = Taldy Suu).

In 42/68 quadrats it was not possible to measure out 50×50 m due to the presence of buildings or other structures. In these cases. smaller areas (mean = 1660·7 m^2^, s.d. = 588·6 m^2^) were measured and the size was recorded, with faecal densities calculated as faeces/100 m^2^. Canid faecal densities ranged from a median of 0·45 faeces/100 m^2^ in Kara Kavak to 1·20 faeces/100 m^2^ in Kashka Suu in May; and from a median of 0·22 faeces/100 m in Sary Mogol to 0·60 faeces/100 m in Kashka Suu in October. The Poisson-Normal GLMM found no evidence that random effects were village specific, nor evidence of any interaction between village and date of visit. The final model including quadrat ID as an overall random effect showed no evidence of overdispersion. There was strong evidence of a significant difference between faecal contamination in Kashka Suu and all other villages (Wald *P*<0·001). Compared to Kashka Suu, the density of faeces in Sary Mogol was 0·46 (95% confidence interval 0·37–0·57); in Taldy Suu 0·57 (0·46–0·70); and in Kara Kavak 0·42 (0·34–0·52). Additionally, there was very strong evidence of a reduction in faecal contamination between visits (Wald *P*<0·001), with the density of faeces in October being 0·53 of that in May (95% CI 0·50–0·56). Fig. 5 shows the crude estimates of the faecal densities amongst the different villages over the two visits.

**Fig. 6.**
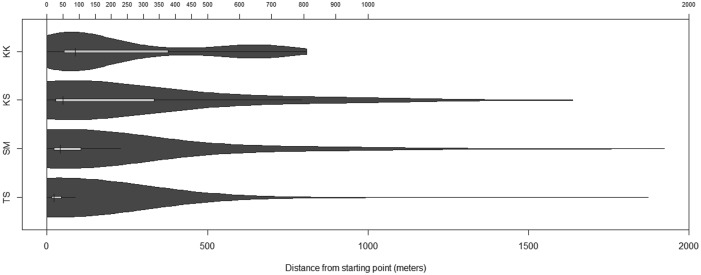
Distances travelled by dogs from each village. The light bar represents the interquartile range, the horizontal black line represent the distances to the ‘inner fence’ (1·5 times the interquartile range), and the vertical black lines represent the median. The dark grey areas represent the probability density.

### CoproELISA results of faecal quadrat samples

In May 2012, a total of 104 faecal samples were collected from the quadrats in the four villages (KK = 24, KS = 28, SM = 28, TS = 24), of which 7 (6·7%) tested positive for *Echinococcus* spp. ELISA positives ranged from 1/28 in Kashka Suu to 3/24 in Taldy Suu, with 1/24 and 2/28 ELISA positives in Kara Kavak and Sary Mogol respectively. In October 2012 a total of 100 ground faecal samples were collected (KK = 24, KS = 24, SM = 28, TS = 24) of which 18 (18%) tested positive. ELISA positives ranged from 2/24 in Kara Kavak to 8/24 in Taldy Suu, with 3/24 and 5/28 ELISA positives in Kashka Suu and Sary Mogol, respectively.

### PCR results of faecal quadrat samples

All 25 ELISA-positive samples that had been collected from the faecal quadrats were analysed for *E. multilocularis, E. granulosus* G1, and *E. granulosus* (sensu lato) using coproPCR. Three samples (1 from KS, 1 from SM, 1 from TS) tested positive for *E. granulosus* (sensu lato), and four samples (1 from KK, 1 from SM and 2 from TS) tested positive for *E. multilocularis*. One of these (a sample collected from a quadrat in Sary Mogol in October) was a mixed infection, testing positive for both *E. multilocularis* and *E. granulosus* (sensu lato). All coproPCR-positive samples were collected in October; all seven ELISA positive samples collected in May were coproPCR negative. The remaining 18 ELISA positive samples that were analysed with coproPCR were PCR negative.

### Arecoline purge data

Of the 20 arecoline purges, eight were scored macroscopically as *Echinococcus* spp. positive in the field. Of these eight positive samples, three also harboured *Taenia* spp. In addition, seven faecal samples were scored macroscopically as *Taenia* spp. positive but *Echinococcus* spp. negative. All 20 purges were analysed using coproELISA. Seven of the eight *Echinococcus* spp. purge-positive samples were also coproELISA positive (five from Kara Kavak and two from Taldy Suu). Additionally, one sample (from Kara Kavak) that had not been scored as *Echinococcus* spp. positive in the field (but was scored as *Taenia* spp. positive) was coproELISA positive. The remaining six *Taenia* spp. purge positives were coproELISA negative. The eight coproELISA positive samples were analysed for *Echinococcus* species using coproPCR. Three of these tested positive for *E. granulosus* G1 using the ND1 primers (Boufana *et al.* 2013), and all eight tested positive for *E. granulosus* (sensu lato) using the Abbasi primers (Abbasi *et al.* 2003; Boufana *et al.* 2008). One sample from Taldy Suu also tested PCR positive for *E. multilocularis* (Boufana *et al.* 2013), indicating a mixed infection. The eight coproPCR-positive samples were sequenced using the Abbasi primers (Beckman Coulter Genomics, Essex, UK). BLAST search gave 99% match to *E. granulosus* (accession number DQ157697) with no specification of genotype. One sample, for which there was sufficient DNA present (from Kara Kavak), was further analysed and sequenced using generic cestode primers (von Nickisch-Rosenegk *et al.* 1999). BLAST search gave 99% match to *E. canadensis* (NCBI accession number AB794685).

### Dog movement data

Four stationary loggers were evaluated simultaneously over a period of 12 hours in the UK (633 recorded points). The distance recorded from the true location for the UK loggers ranged from 0 m to 206 m, with 95% of recorded locations for each logger being less than 45 m. The stationary logger left for 3.5 h in Kyrgyzstan (total 40 points), recorded a difference of 0 to 32 m, with a median of 5 m.

Of the two dynamic recordings made in Kyrgyzstan (a total of 155 points), the median difference from the true location was 16 m, with 95% of readings being within 70 m of the true value. The dynamic recordings made in the UK (35 points) showed a median difference of 31 m with 95% of readings being within 90 m of the true value.

Table 2 shows the median 95% CHP areas and distances travelled from the start location for those dogs monitored in each village. Dogs with less than 50 points recorded were excluded from analysis. A significant difference in distance travelled was found between villages (Kruskal Wallis test *P*<0·001) which was present when each individual village was compared with each other using a pairwise Wilcoxon test with Holm–Bonferroni correction (*P*<0·001 in all cases). The same overall effect was also found when individual dogs were accounted for by comparing the median distance travelled (Kruskal Wallis test *P* = 0·004), although with this analysis there was only a significant difference found between Taldy Suu and Kara Kavak or Kashka Suu (Wilcoxon with Holm–Bonferroni correction *P* = 0·02 and *P* = 0·01, respectively). No difference was found in the size of the home ranges between villages (*P* = 0·13). There was also no difference in the size of the home range between males and females, either overall (Wilcoxon rank sum, *P* = 0·50) or within those villages with both sexes represented (SM *P* = 0·53; KS *P* = 0·25). There was also no difference in the median distance travelled (per dog) according to sex (overall Wilcoxon rank sum *P* = 0·85; SM only *P* = 0·41; KS only *P* = 0·25). Village areas based on MCP methods are shown in Table 2. Of all 37 dogs studied, 22 (59%) left the village boundary at least once during monitoring. No difference was found in this proportion between different villages (Chi square test *P* = 0·73).

**Table 2.** Dog movements and home range sizes for the dogs monitored in this study. (Figures in brackets relate to the bootstrapped confidence interval for the median.)

Village	Village area (km^2^)	Number of points	Number of dogs monitored	Median home range (m^2^)	Median of median distance travelled per dog (m)
SM	3·32	1494	11	22 650	39 (31–84)
TS	1·63	2459	13	15 700	20 (20–29)
KS	1·16	1637	10	37 490	46 (28–308)
KK	0·81	666	3	29 730	62 (58–629)
*Overall*	*–*	*6256*	*37*	*22* *650*	*35 (28–48)*

## DISCUSSION

Echinococcosis is a national public health concern in Kyrgyzstan (Torgerson *et al.* 2002; Usubalieva *et al.* 2013). However, no studies of canine echinococcosis have been undertaken in the Alay Valley of Osh oblast in the south-west of the country. Furthermore, no information exists in Kyrgyzstan about environmental faecal contamination and behaviour of dogs in relation to transmission of *Echinococcus* spp.

The faecal samples collected and analysed from arecoline-purged dogs confirmed that *Echinococcus* spp. are present in domestic dogs in the Alay Valley. This expands the known distribution of canine echinococcosis in southern Kyrgyzstan (Ziadinov *et al.* 2008). The ELISA-positive arecoline purges (8/20) were all tested using coproPCR and the results show that *E. granulosus* sensu lato (8/8), including *E. granulosus* G1 (3/8), as well as *E. canadensis* (1/8), and *E. multilocularis* (1/8) are present in domestic dogs in the Alay Valley. With regard to environmental contamination, three of the ELISA-positive faecal samples collected from the faecal quadrats (25/204) tested DNA positive for *E. granulosus* (sensu lato), and four samples tested DNA positive for *E. multilocularis*. For 72% of ELISA-positive samples however, the coproPCR analysis (for *E. granulosus* and *E. multilocularis*) yielded a negative result. This is likely due to the fact that many of the ground faecal samples collected from the quadrats were not fresh, and DNA in faeces is known to degrade over time unless preserved properly (e.g. Olson *et al.* 2005). Although these data confirm the presence of *Echinococcus* spp. in dogs in the Alay Valley, the data presented here are neither sufficient to infer canine infection rates with *Echinococcus* spp. nor to assess seasonality of canine echinococcosis. Further surveys are currently being undertaken by us to assess canine echinococcosis in the owned dog populations in the Alay Valley more accurately.

Between 38·0 and 74·4% of households in the four communities surveyed in the Alay Valley owned at least one dog. Male dogs were more commonly kept than females, as is often the case in rural communities (e.g. Butler and Bingham, 2000), and this may be related to males being seen as better guard/sheep dogs, or it may be due to people not wanting to deal with pups. Questionnaire analysis revealed that almost all dogs were free to roam, with very few dogs being leashed. In addition, the dog population in all four communities appeared to have a high turnover. The local municipality in Gulcha (district administrative capital) arranges for dogs to be culled at least once a year in order to control dog population numbers (Akjol Tagaibekov, local veterinarian, personal communication), and we observed a decline in dog numbers between May and October. Although many people acquired new dogs between visits, few of these did so to replace dogs which were lost or died. As such, based on current observations, the dog population in these Kyrgyz communities appears to be quite dynamic, with changes in dog numbers and dog ownership.

Contamination with dog faeces was high in all four villages, with an overall faecal density of 77·6 and 41·3 faeces/hectare in May and October, respectively; this is higher than the faecal contamination reported in highly endemic *Echinococcus* spp. rural communities in western China (Vaniscotte *et al.* 2011). The overall density of dog faecal contamination in the communities was significantly lower in October than in May. This may be due to the dog culling that took place before and during October, or that faeces degrade faster in the warmer months between May and October than in earlier months. Based on these two sampling times, however, we cannot conclude that environmental contamination is always higher during spring than autumn. Dog culling may take place twice a year and be at different times of the year (Akjol Tagaibekov, local veterinarian, personal communication), and this will clearly affect dog faecal densities. Dog faeces present a risk to humans as *Echinococcus* spp. eggs may survive in the environment for hundreds of days (Veit *et al.* 1995). The majority of dogs in these communities were free roaming, and as a result even gardens belonging to families that did not own dogs or areas surrounding dog-free households were often contaminated with dog faeces (F. van Kesteren, personal observation). Faecal contamination was also notably higher in Kashka Suu than in the other three villages, probably due to a higher dog density in this village.

The stationary and dynamic recordings by the iGotU^®^ units suggest that these GPS loggers can be used to monitor dog movements with reasonable accuracy. Although the battery life of the iGotU^®^ units was limited and several units switched off prior to collection, the iGotU^®^ units have several advantages over conventional GPS animal monitoring units. These benefits include a very small size (20 g), frequent recording capacities and affordability. As in community dogs in western China (Vaniscotte *et al.* 2011), dogs mostly stayed within a few hundred metres of their owners’ homes (median 11–931 m), with median home ranges between 15 700–37 490 m^2^. Dogs also roamed up to 2 km away from their owners’ home and most (59%) left the village boundary. Furthermore, our estimates of dog movements are probably conservative as we could include only dogs that were present and tame enough to be handled (i.e. those dogs that accompany livestock to pasture during the day were not included and aggressive dogs were not included but may have been more active than tamer dogs). There were significant differences in the distances travelled by dogs between different villages, with dogs in Taldy Suu generally travelling shorter distances than those in other villages. Although there was no evidence of any significant difference in the sizes of their core home ranges, this may be a result of the relatively small sample size as the general trend in home range size was similar to that of median distance travelled (Table 2). In addition, although previous studies have found that male dogs generally move further than females (e.g. Vaniscotte *et al.* 2011), no evidence of a sex difference was found here. Although over 88% of people in all villages reported never feeding their dogs offal and only few people (5–39% per village) reported seeing their dogs eat rodents, the fact that dogs roamed freely and moved outside of their communities meant that people could not be sure of what their dogs were eating. Dogs were observed eating offal on several occasions (F. van Kesteren, personal observation) and are likely to consume small rodents in or around villages.

Kyrgyzstan became independent around the time of the collapse of the Soviet Union in 1991 and has since been through considerable political and economical changes (Torgerson *et al.* 2002). During Soviet times, the rearing of sheep (the primary intermediate host for *E. granulosus*) took place on large collectivized farms, slaughter was undertaken in large slaughterhouses under veterinary inspection and treatment of farm dogs with praziquantel every four months was compulsory (Torgerson *et al.* 2002), contributing to relatively low levels of human echinococcosis (Torgerson *et al.* 2002, 2006). In contrast, since independence collective farms have broken up into small farms, home slaughter has increased, and the dog population has grown (Torgerson *et al.* 2006), which has been implicated as the cause of higher rates of human echinococcosis (Torgerson *et al.* 2002, 2006). Our data show that dogs are common in rural communities in the Alay Valley in southern Kyrgyzstan. The majority of dogs roamed freely and may roam several kilometres away from their owners’ home, thus being able to scavenge offal and consume rodents, putting them at risk of infection with *E. granulosus, E. canadensis* and *E. multilocularis*. The free-roaming dogs also defaecate wherever they are, thus putting people in the community at a potential risk of infection with *Echinococcus* spp.

We have now confirmed the first reports of *E. multilocularis, E. granulosus* G1 and *E. canadensis* in dogs in the Alay Valley, Osh oblast, Kyrgyzstan. Attempts to control and even eliminate echinococcosis have been carried out in several different locations, with differing degrees of success (Gemmell *et al.* 1986; Craig and Larrieu, 2006). The World Bank has recently proposed an *Echinococcus* control programme for Kyrgyzstan, which includes providing anthelminthics for dogs (World Bank, 2011), and this programme was already underway in October 2012 (Akjol Tagaibekov, local veterinarian, personal communication). However, hydatid control programmes will benefit from being informed by an understanding of dog population size, basic dog ecology and dog behaviour. Collecting data such as that presented here can improve the efficacy of intervention programmes. Further studies to gain knowledge on dog population turnover and infection and re-infection rates will be beneficial, especially to determine optimal cost-benefits of dog dosing schedules.
